# Trajectory patterns and influencing factors of depression due to child bereavement among older adults in China: a 5-year longitudinal study

**DOI:** 10.3389/fpubh.2025.1548256

**Published:** 2025-05-09

**Authors:** Li Chen, Zhangyi Wang, Qiaoyan Wu, Xiaochun Tang

**Affiliations:** ^1^School of Nursing, Hunan Normal University & Affiliated Hengyang Central Hospital, Changsha, Hunan, China; ^2^School of Nursing, Kiang Wu Nursing College of Macao, Macao, Macao SAR, China

**Keywords:** child bereavement, depressive symptoms, developmental trajectory, latent growth mixture model, older adults

## Abstract

**Objective:**

As a result of the aging of the world population, a high number of older adults lose their children during their lifetime. Depression due to child bereavement is a significant psychological problem. Therefore, it is necessary to investigate the trajectories of depressive symptoms associated with child bereavement among older adults in China and determine the influencing factors.

**Methods:**

In this study, data from the China Health and Retirement Longitudinal Study were used as the longitudinal data, and 284 women and 117 men aged over 60 years were included. A latent growth mixture model was used to identify trajectory patterns in depression due to child bereavement over time. Multivariate logistic regression analysis was used to determine the influencing factors.

**Results:**

Four trajectory patterns of depressive symptoms associated with child bereavement were identified: a low depression rapidly increasing group (12.0%), a high depression rapidly declining group (12.1%), a high depression slowly increasing group (23.1%), and a low depression stable group (52.8%). The findings of the multivariate logistic regression analysis showed that residence, sleep status, satisfaction with life, and self-report of health were related to the trajectory patterns of depressive symptoms among the participants.

**Conclusion:**

This study revealed heterogeneity in changes in depressive symptoms among older adults with child bereavement in China. The government and medical institutions should consider these trajectory patterns of depression and adopt individualized support measures based on the characteristics of different groups.

## 1 Introduction

Aging is a critical public health issue worldwide (1). In the 2020 China Census, the proportion of older adults aged 60 years and above was 18.70%, an increase of 5.44 percentage points compared with that in 2010 (2). In the context of global population aging, the concept of “healthy ageing,” as proposed by the World Health Organization (WHO), is especially important (3, 4). However, as the older adult population increases, a high number of older adults may lose their children during their lifetime (5). Child bereavement can be the most distressing event for older parents, especially in China. In traditional Chinese culture, children play an important role in the family. They not only offer emotional and material support to their older adult parents but also fulfill their filial support obligations. Moreover, children are regarded as the most crucial spiritual sustenance for the Chinese older adult (5, 6). According to a report (7), it is estimated that 10 million families will experience child bereavement in China by 2035. Due to the physical frailty factors associated with the aging process, the mental health of older adults is more likely to be influenced by loss (8, 9), and the risk of developing depression may be high (10). Studies (7, 11) have shown that the loss of a child can be more persistent and intense in older parents than other types (such as loss of spouse) of bereavement-related depression, and it may also induce the persistence of abnormalities in the inferior parietal cortex. Therefore, losing children can result in a huge psychological shock for older adults. Thus, the early prediction of and interventions for influencing factors will be of great significance in reducing the prevalence of depression related to child bereavement.

In older adults, depression after losing their children, which manifests as emotional and psychological responses such as helplessness, sadness, self-blame, and self-guilt, can change over time (12). Studies have shown that approximately 34% of mothers and 35% of fathers suffer from moderate-to-severe depression 3 to 5 years after they lose their child (10). In addition, depression related to child bereavement is a chronic stressor in older adults, exerting cumulative and detrimental effects on their psychological well-being (13). However, some studies have found heterogeneity in older adults in terms of the depression status (14, 15). Some older adults who have a brief but intense experience of depression show a gradual decline in depressive symptoms, whereas others show progressively increasing symptoms of depression (16). Research has found that the trends in depression caused by child bereavement are different at different periods. For example, some older adults experience less intense depression in the short term after the loss of a child, whereas others experience significant depression within 18 months after the loss (17). These results suggest that longitudinal trajectories in child-bereavement-related depression are heterogeneous (18).

The majority of the existing studies have focused only on the changes in depressive symptoms among young or middle-aged parents with child bereavement, but without taking into account the trajectory patterns in older adults with child bereavement (18, 19). In addition, since the trajectory patterns of child-bereavement-related depression change in individuals over time (20), changes in depressive symptoms can be regarded as a stage-sequential process (21). This observation points to the specific pattern of bereavement-related depression in older adults who have lost a child.

Currently available studies have limitations in understanding the evolution of bereavement-related depression. The majority of these studies rely on cross-sectional surveys or qualitative research. Cross-sectional studies (22–24) only capture a single-point depressive state and do not consider long-term changes. Qualitative research, though insightful, cannot systematically quantify these temporal shifts. Furthermore, traditional longitudinal data analysis methods (18) assume a homogeneous population and overlook individual differences, which leads to inaccurate conclusions. In contrast, the latent growth mixture model (LGMM) (25), which is designed for longitudinal data, can reveal heterogeneity in growth trajectories. By estimating the parameters for different latent classes, it identifies distinct change patterns. The present study uses five-year follow-up data from a representative older adult Chinese sample. Using the LGMM, it aims to uncover trajectory patterns of depression in older adult parents who have lost their children and fill gaps in the current literature.

## 2 Methods

### 2.1 Data source

**Figure 1:**
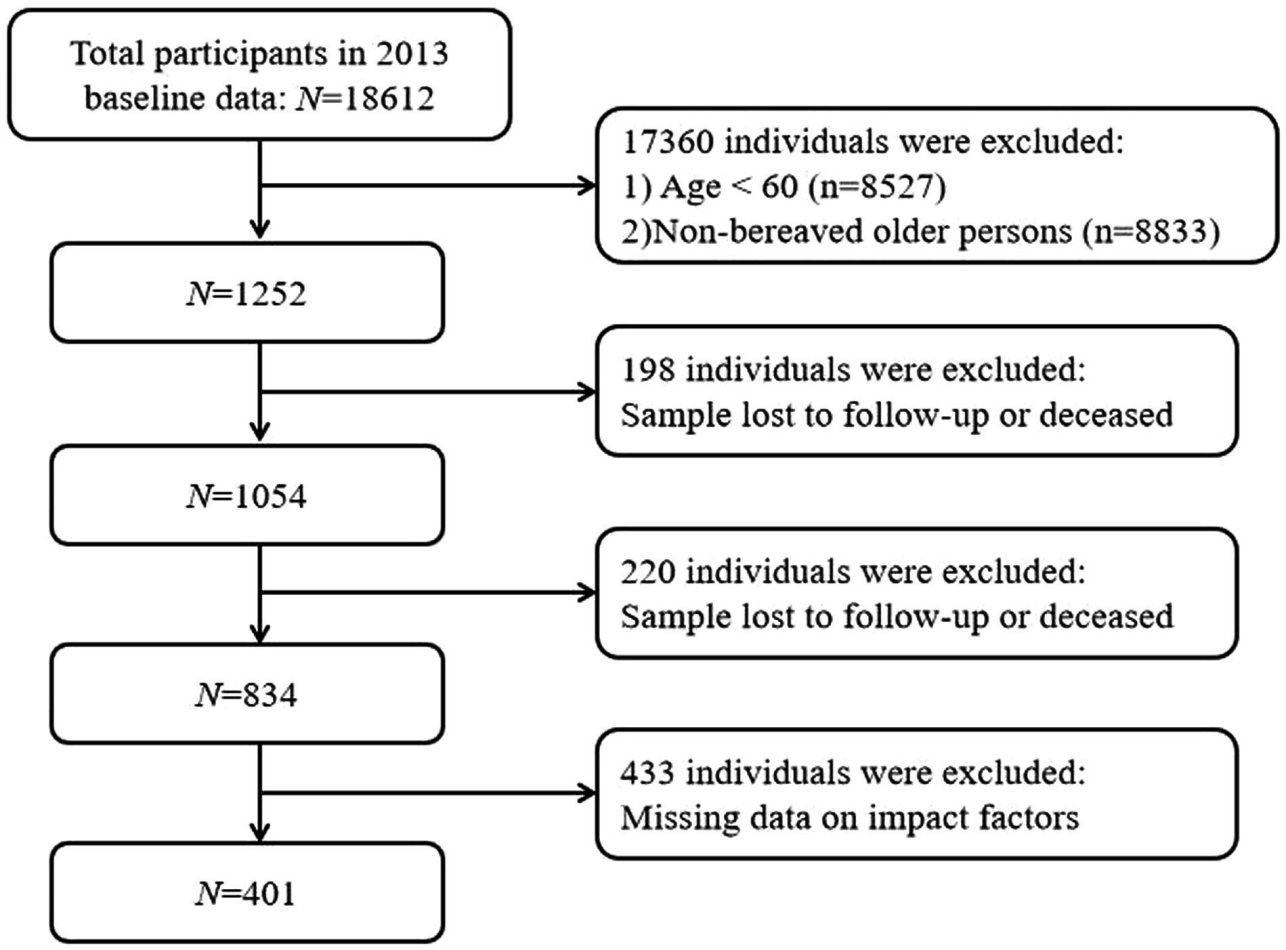
The study flow chart.

The data were acquired from the China Health and Retirement Longitudinal Study (CHARLS) database, which is a high-quality public database that includes information on middle-aged and older residents in China. Currently, the CHARLS database has been used in various research fields, such as economics, demography, public health, and nursing. The data used in this study were drawn from three waves of surveys of the CHARLS database conducted in 2013, 2015, and 2018. The inclusion criteria were as follows: (1) baseline age≥60 years; (2) indicated child bereavement (loss of at least one child) in the baseline survey in 2013; and (3) participants who completed all three phases of the survey. The exclusion criteria were as follows: (1) missing depressive symptom scores in one phase or more and (2) missing data on other influencing factors. In 2013, the total number of older adult individuals with child bereavement was 1,007. When the survey was conducted in 2015, 1,054 individuals were successfully followed up, and 198 were lost to follow-up or had died. In 2018, 834 individuals were followed up again, and 220 were lost to follow-up or had died. The total number of samples lost to follow-up or dead was 418, which accounted for 33.3%. In total, 401 participants aged 60 to 93 years were included in this study, with 284 (70.80%) being female and 117 (29.20%) being male. The sample selection process is presented in detail in Figure 1. A comparison of baseline characteristics was carried out to verify the representativeness of the sample. Specifically, the demographic characteristics (age, gender, educational level, residence, and marital status) of the 401 participants were compared with those of the total number of older adult individuals who had experienced child bereavement in 2013. The findings of this comparison showed that there were no statistically significant differences between the two groups (all *p* > 0.05). Further details are presented in Table 1.

**Table 1:** Comparison of demographic characteristics.

Demographic characteristics	Initial Sample of the older adult with child Bereavement (*n* = 401)	Final included sample (*n* = 1,252)	Statistic	*p*-value
Age (years, xˉ ± s)	70.87 ± 8.31	71.92 ± 10.28	*t* = −1.871	0.062
Gender (female, %)	70.80(284)	66.45(832)	*χ*^2^ = 2.644	0.104
Education level (illiteracy, %)	48.38(194)	49.84(624)	*χ*^2^ = 0.259	0.611
Residence (rural, %)	71.07(285)	69.41(869)	*χ*^2^ = 0.399	0.528
Marital status (married, %)	72.31(290)	75.96(951)	*χ*^2^ = 2.150	0.143

### 2.2 Dependent variable

Bereavement-related depressive symptoms in older adults were quantified using the 10-item version of the Centre for Epidemiologic Studies Depression Scale (CES-D-10) (26). CES-D-10 was derived from the 20-item Epidemiological Studies Depression Scale. The 10 items in CES-D-10 included three depression items, five physical symptom items, and two positive emotion items. Each item had four options: “Rarely or none of the time (<1 day),” “Some or a little of the time (1–2 days),” “Occasionally or a moderate amount of time (3–4 days),” and “Most or all of the time (5–7 days).” Each item was scored on a scale of 0 to 3, with the exception of items 3 and 5, which were reverse-scored. The total score ranged from 0 to 30, and participants who scored ≥ 10 points were considered having depressive symptoms. The Chinese version of this scale achieved a Cronbach’s alpha value of 0.813 when administered to the older adult population in China (27).

### 2.3 Independent variables

Data regarding influencing factors were collected in 2013. Fifteen factors were included: (1) age; (2) gender; (3) residence; (4) marital status; (5) educational level; (6) smoking; (7) drinking; (8) number of living children; (9) number of deceased children; (10) disability; (11) sleep restlessness; (12) life satisfaction; and (13) difficulty with any one of the instrumental activities of daily living (IADLs, namely doing household chores, cooking, shopping, managing money, and taking medications) (28); (14) chronic comorbidity; (15) self-report of health.

### 2.4 Statistical analysis

Statistical analysis of the data was carried out using SPSS 27.0 software. The LGMM analysis was conducted using Mplus 8.3 software. The best-fit model was selected by comparing fit indexes across multiple models, taking into account both practical significance and statistical indicators. This approach ensured that the selected model not only statistically fit the data well but also had meaningful implications for understanding the latent growth patterns. The fitting indices for this model verification were as follows: (1) Akaike information criterion (AIC), Bayesian information criterion (BIC), and sample-adjusted Bayesian information criterion (aBIC); (2) entropy; and (3) test statistics: Lo–Mendell–Rubin likelihood ratio test (LMRT) and bootstrapped likelihood ratio test (BLRT) (29). The lower the values of AIC, BIC, and aBIC, the better the fitting effect of the model. The closer the entropy value is to 1, the more accurate the classification of the model. Both LMRT and BLRT reached a significant level (*p* < 0.05), which indicated that the model with k categories showed a better fitting effect than the model with k-1 categories. To ensure clinical significance, each category should include at least 5% of the participants (30). Chi-square test was used to investigate the relationship between sociodemographic and clinical variables in different subgroups. Multivariate logistic regression, with potential categories of depression among older individuals who have lost a child as the dependent variable, was used to identify the influencing factors for different potential categories of depression among this population. Statistical significance was set at *p* < 0.05, and all *p* values were two-sided.

### 2.5 Ethical approval

Approval for the CHARLS data was granted by the Ethics Review Committee of Peking University, with the reference number IRB00001052-11015.

## 3 Results

### 3.1 Identification of the trajectory patterns of depressive symptoms of child bereavement among older adults

This study analyzed the trajectory patterns of three-wave CES-D-10 scores in individuals with child bereavement. The participants’ overall CES-D-10 scores were 9.88 ± 6.574, 10.48 ± 7.182, and 11.08 ± 7.325 at wave 1, wave 2, and wave 3, respectively. The fit indexes of the LGMM were used to identify three trajectory groups. Compared with other models, model 4 showed lower AIC and BIC values. As shown in Table 2, in model 4, both LMRT and the BLRT were statistically significant. Therefore, model 4 was identified as the best model.

**Table 2:** Fitting of the LGMM of depressive symptoms of older adults with child bereavement in China.

GMM	LL	*AIC*	*BIC*	*aBIC*	*Entropy*	*P (LMRT)*	*P (BLRT)*	Probability (%)
1C	−3889.667	7795.335	7827.286	7801.902	–	–	–	1.000
2C	−3864.272	7750.544	7794.477	7759.573	0.749	*<*0.001	*<*0.001	32.9/67.1
3C	−3860.686	7749.372	7805.288	7760.865	0.755	0.4323	0.3333	03.5/31.2/65.3
4C	−3845.541	7725.081	7792.979	7739.036	0.763	0.0300	<0.001	10.2/11.5/23.9/54.4
5C	−3839.954	7719.909	7799.788	7736.326	0.801	0.4483	0.0300	2.5/50.1/25.2/14.0/8.2

aBIC, sample-size-adjusted Bayesian information criterion; AIC, Akaike information criterion; BIC, Bayesian information criterion; BLRT, bootstrapped likelihood ratio test; LL, log likelihood; LMRT, Lo–Mendell–Rubin adjusted likelihood ratio test.

**Figure 2:**
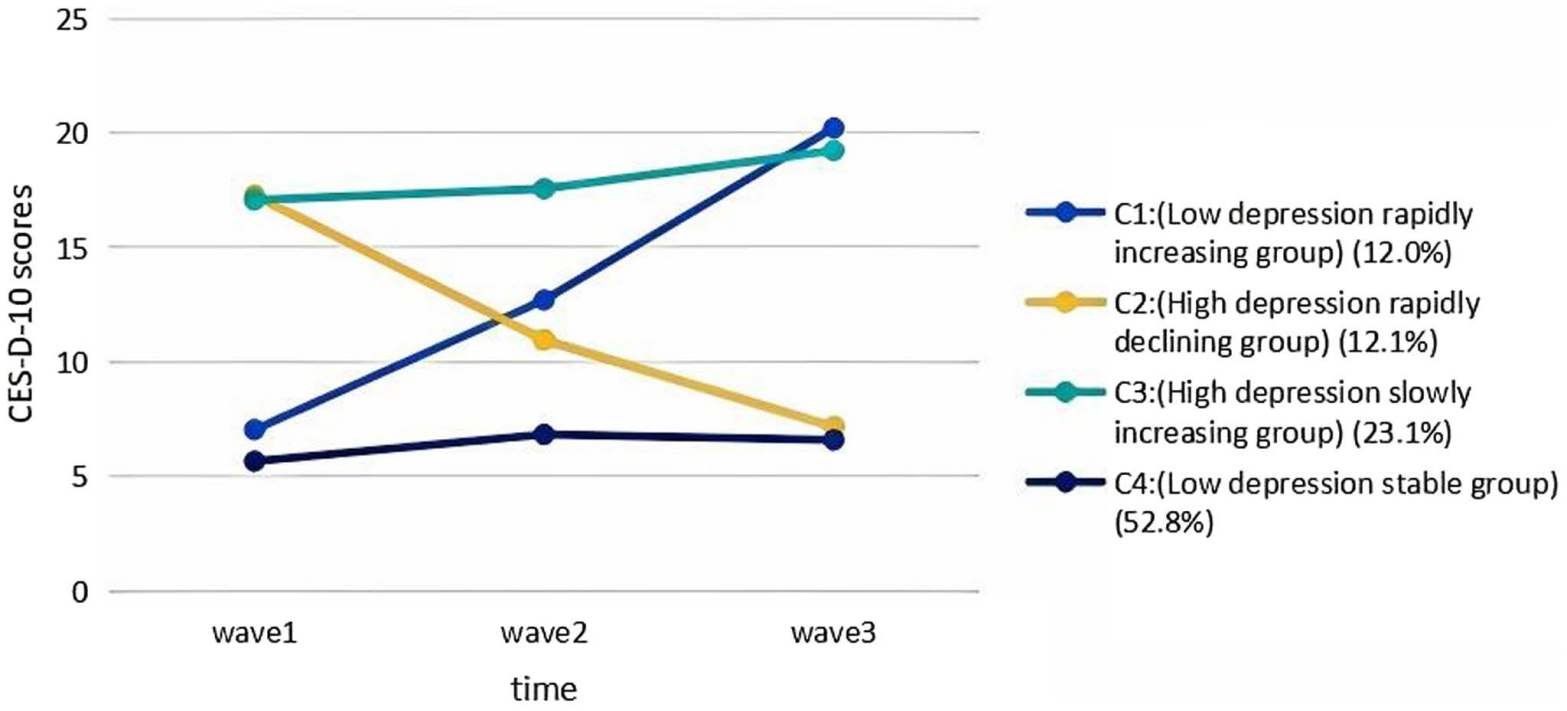
The trajectories of depressive symptoms among bereaved older adults in China.

Using model 4, a trajectory plot was constructed with CES-D-10 scores as the vertical axis and wave 1 to wave 3 as the horizontal axis. This plot categorized the trajectory patterns of depressive symptoms among older Chinese adults who have experienced the loss of a child into four distinct groups, as shown in Figure 2:

1. Low depression rapidly increasing group (C1): This group had a low initial score (7.05) (*I* = 7.846, *p <* 0.05), and depressive symptoms improved effectively over time (*S* = 5.925, *p <* 0.05). It consisted of 41 older adults, accounting for 12.0% of the older adults.
2. High depression rapidly declining group (C2): This group had a high initial score (17.28) (*I* = 16.532, *p*<0.05), and depressive symptoms declined rapidly over time (*S* = -4.600, *p*<0.005). It consisted of 46 older adults (12.1%).
3. High depression slowly increasing group (C3): The mean intercept and mean slope were 16.673 (*p <* 0.05) and 0.925 (*p >* 0.05), respectively, and the initial score (17.08) of this group was high (*I* = 16.673, *p <* 0.05), with an overall increasing trend. In addition, depression symptoms did not significantly improve over time (*S* = 0.925, *p >* 0.05), and this group consisted of 96 older adults (23.1%).
4. Low depression stable group (C4): This group had the lowest initial score (5.67) (*I* = 5.844, *p <* 0.05), and depressive symptoms remained stable over time (*S* = 0.0445, *p <* 0.05). This group had 218 older adults (52.8%).

The significant level of variance in the intercepts between the four patient groups (*p <* 0.05) indicates that there are individual differences in the initial levels of depressive symptoms between older adult individuals who have experienced the loss of a child. In contrast, the lack of significant variance in the slopes between the four groups suggests that there are no individual differences in the rate of change of depressive symptoms among these older adult individuals.

### 3.2 Single-factor analysis of influencing factors for trajectory patterns of depressive symptoms in older adults with child bereavement

Using these latent classes as grouping variables, the baseline characteristics of older adults with child-bereavement-related depression belonging to different trajectory categories were compared. The results showed that there are statistically significant differences between the four groups (*p* < 0.05) in the distributional comparisons of gender (*χ^2^* = 11.823, *p* = 0.008), residence (*χ^2^* = 16.904, *p* = 0.001), sleep restlessness (*χ^2^* = 16.904, *p* < 0.001), life satisfaction (*χ^2^* = 27.985, *p* < 0.001), difficulty with IADLs (*χ^2^* = 50.953, *p* < 0.001), number of chronic diseases suffered (*χ^2^* = 20.806, *p* = 0.014), and self-report of health (*χ^2^* = 22.677, *p* < 0.001). Further information is presented in Table 3.

**Table 3:** Comparison of general data of different depression trajectory groups (*N* = 401).

Variables	Code	C1 (*n* = 41)	C2 (*n* = 46)	C3 (*n* = 96)	C4 (*n* = 218)	*χ* ^2^	*p*-value
Age at baseline, years						4.342	0.227
60–69	1	24 (58.5)	24 (52.2)	61 (63.5)	112 (51.4)		
≥70	2	17 (41.5)	22 (47.8)	35 (36.5)	106 (48.6)		
Gender						11.823	0.008
Male	0	8 (19.5)	11 (23.9)	19 (19.8)	79 (36.2)		
Female	1	33 (80.5)	35 (76.1)	77 (80.2)	139 (63.8)		
Residence						16.904	0.001
City	1	12 (29.3)	12 (26.1)	13 (13.5)	79 (36.2)		
Rural	2	29 (70.7)	34 (73.9)	83 (86.5)	139 (63.8)		
Marital status						1.434	0.698
Married	1	28 (68.3)	36 (78.3)	71 (74.0)	155 (71.1)		
Widowed/single	2	13 (31.7)	10 (21.7)	25 (26.0)	63 (28.9)		
Educational level						11.022	0.088
Illiterate	1	25 (61.0)	21 (45.7)	47 (49.0)	101 (46.3)		
Primary school or lower	2	16 (39.0)	22 (47.8)	40 (41.7)	92 (42.2)		
Junior high school and above	3	0 (0.0)	3 (6.5)	9 (9.3)	25 (11.5)		
Smoking						2.365	0.500
No	0	35 (85.4)	37 (80.4)	82 (85.4)	172 (78.9)		
Yes	1	6 (14.6)	9 (19.6)	14 (14.6)	46 (21.1)		
Drinking						4.964	0.174
No	0	32 (78.0)	39 (84.8)	70 (72.9)	152 (69.7)		
Yes	1	9 (22.0)	7 (15.2)	26 (27.1)	66 (30.3)		
Number of living children						4.550	0.603
0–2	1	17 (41.5)	11 (23.9)	30 (31.3)	81 (37.2)		
3–5	2	20 (48.8)	29 (63.0)	54 (56.3)	115 (52.8)		
≥6	3	4 (9.7)	6 (13.1)	12 (12.4)	22 (10.0)		
Number of deceased children						5.394	0.145
1	1	33 (80.5)	35 (76.1)	69 (71.9)	181 (83.0)		
≥2	2	8 (19.5)	11 (23.9)	27 (28.1)	37 (17.0)		
Disability						4.876	0.181
No	0	36 (87.8)	43 (93.5)	88 (91.7)	209 (95.9)		
Yes	1	5 (12.2)	3 (6.5)	8 (8.3)	9 (4.1)		
Sleep restlessness, days						102.870	<0.001
<1	1	21 (51.2)	7 (15.2)	15 (15.6)	130 (59.6)		
1–2	2	6 (14.6)	5 (10.9)	13 (13.5)	37 (17.0)		
3–4	3	10 (24.4)	13 (28.3)	21 (21.9)	28 (12.8)		
5–7	4	4 (9.8)	21 (45.6)	47 (49.0)	23 (10.6)		
Life satisfaction						27.985	<0.001
Not satisfied	0	5 (12.2)	13 (28.3)	24 (25.0)	14 (6.4)		
Satisfied	1	36 (87.8)	33 (71.7)	72 (75.0)	204 (93.6)		
Difficulty with IADLs						50.953	<0.001
No	0	18 (43.9)	22 (47.8)	39 (40.6)	135 (61.9)		
Yes	1	23 (56.1)	24 (52.2)	57 (59.4)	83 (38.1)		
Number of chronic diseases suffered						20.806	0.014
0	1	10 (24.4)	2 (4.3)	14 (14.6)	47 (21.6)		
1	2	10 (24.4)	15 (32.6)	17 (17.7)	62 (28.4)		
2	3	8 (19.5)	16 (34.8)	23 (24.0)	52 (23.9)		
≥3	4	13 (31.7)	13 (28.3)	42 (43.7)	57 (26.1)		
Self-report of health						47.215	<0.001
Healthy	1	7 (17.1)	10 (21.7)	9 (9.4)	73 (33.5)		
General	2	24 (58.5)	17 (37.0)	41 (42.7)	109 (50.0)		
Fragile	3	10 (24.4)	19 (41.3)	46 (47.9)	36 (16.5)		

### 3.3 Logistic regression analysis of influencing factors for trajectory patterns of depressive symptoms in older adults with child bereavement

Logistic regression was conducted using the results of the trajectory category analysis as the dependent variable and all significant variables from the single-factor analysis as independent variables. The results revealed that in the comparison of C1 with the reference group C3, city dwellers and those with sleep restlessness for <1 day, 1–2 days and 3–4 days were more likely to be categorized as C1. Similarly, when comparing C2 with the reference group C3, city dwellers were more likely to be categorized as C2. In addition, when comparing C4 with the reference group C3, city dwellers; those with sleep restlessness of <1 day, 1–2 days, and 3–4 days; and those with self-reported good health were more likely to be categorized as C4. Older adults who were not satisfied with life were more likely to be classified as C3 (all *p* < 0.05). Further details about logistic regression analysis are presented in Table 4.

**Table 4:** Multivariate logistic regression analysis of the trajectory of depressive symptoms of older adults with child bereavement (*N* = 401).

Groups	Related factors	*β*	SE	Wald *χ*^2^	*p*-value	OR	95% CI
C1 vs. C3	City	1.535	0.507	9.159	0.002	4.643	1.718–12.549
	Sleep restlessness (<1 day)	3.024	0.653	21.442	<0.001	20.582	5.722–74.037
	Sleep restlessness (1–2 days)	1.684	0.737	5.219	0.022	5.387	1.270–22.844
	Sleep restlessness (3–4 days)	2.063	0.677	9.300	0.002	7.871	2.090–29.639
C2 vs. C3	City	0.945	0.474	3.981	0.046	2.574	1.017–6.514
C4 vs. C3	City	1.859	0.403	21.321	<0.001	6.419	2.916–14.133
	Sleep restlessness (<1 day)	2.947	0.433	46.312	<0.001	19.054	8.153–44.527
	Sleep restlessness (1–2 days)	1.656	0.460	12.976	<0.001	5.236	2.127–12.890
	Sleep restlessness (3–4 days)	1.389	0.437	10.087	0.001	4.012	1.702–9.457
	Satisfied with life (not satisfied)	−1.321	0.442	8.934	0.003	0.267	0.112–0.635
	Self-report of health (healthy)	1.704	0.509	11.198	0.001	5.496	2.026–14.909

The reference group: residence, rural; sleep restlessness, 5–7 days; satisfied with life, satisfied; self-report of health, fragile; group, C3.

## 4 Discussion

### 4.1 Analysis of the trajectory of change in depressive symptoms in older adults with child bereavement

This study categorized the trajectory of depressive symptoms in older people with child bereavement into four categories: a low depression rapidly increasing group (12.0%), a high depression rapidly declining group (12.1%), a high depression slowly increasing group (23.1%), and a low depression stable group (52.8%), as determined by the LGMM. This suggests the heterogeneity of depressive symptoms in older adults with child bereavement. This finding serves as a guideline to future researchers, emphasizing the need for a nursing assessment system with group heterogeneity and individual heterogeneity.

The proportion of older adults in the C1 group was 12.0%, who had increasingly obvious symptoms of depression, which is consistent with the studies of Pohlkamp et al. (31) and D'Epinay et al. (32). In this study, older parents with bereavement experienced the loss of their most beloved children, and in the early stage of the loss, they may experience shock and even deny the truth. However, as reality continued to hit them, they gradually experienced an emotional breakdown and an increased sense of isolation. In traditional China, first, others may sympathize with the older parents with child bereavement, but over time, these parents may suffer from strange looks, be regarded undesirable, or even be bullied. In China, it is considered an individual tragedy to have no surviving child because the Chinese believe that without a surviving child, there are no descendants and they cannot inherit the bloodline, which is considered an act of shame to the ancestors (33). Therefore, in older adults with child bereavement, depressive symptoms gradually worsen and have lasting effects, even affecting their physical health, consistent with the findings of a previous study (32). These older adults are in the most vulnerable situation as they are older (mean age = 68.46 years), live in rural areas (70.7%), and have a low level of education (61% illiterate and 39% with primary school education or lower). These data indicate that older adults with child bereavement are less likely to have high incomes and receive pensions. Therefore, providing effective social support and promoting the idea of a new era of parenting can contribute to mitigating some adverse effects of the loss among older individuals.

In contrast to the C1 group, the depression level in older adults in the C2 group decreased rapidly over time, and the proportion of those with a significant reduction in depression levels in the C2 group accounted for 12.1%. This trajectory model has long been confirmed in the studies (34, 35), and it even predicts that 7–9 years after bereavement, depression levels of these parents are similar to those of parents with no bereavement (34). Over time, the majority of them gradually recovered from their child-bereavement-related depression through having surviving children, receiving social support, and actively participating in social activities (36). Thus, their psychological condition reached a relatively stable stage with no prolonged grief disorder or post-traumatic stress disorder. This indicates that their depressive symptoms of losing a child had decreased (34). Nevertheless, it is important to emphasize that despite a decline in bereavement-related depressive symptoms, routine mental health assessments and social support should continue, and new stressors that these older adults may encounter should be investigated. Encouraging support and involvement from community, friends, and family is advised, and social volunteering also helps (21). This service is grief-recovery-oriented, which enriches their social networks, improves life satisfaction, and enables them to regain control of their lives. In addition, it is crucial to provide professional and high-quality counselling to these older adults to prevent grief rumination (37).

The proportion of older adults in the C3 group was 23.1%. These older adults had high CED-S-10 scores, indicating no improvement in depressive symptoms, which is in line with the study of Lykke et al. (10). These older adults experienced a multifaceted attack on physical, psychological, and life support after a child loss, such as increased incidence of chronic diseases, loss of their main source of caregiving, termination of family lineage, loneliness, and prolonged grief, resulting in depression. Therefore, they found it difficult to accept the loss of their children and had persistent bereavement-related depressive symptoms, which is consistent with previous studies (33, 38). The concept of “child-rearing for old age” is deeply rooted in Chinese culture, and when older people are confronted with having no child after the loss of their only child, their risk of depression is high and persistent (33, 39). Within 3 to 5 years of losing a child, if older adults show poor psychological resilience, they become more vulnerable to stressors, which in turn may contribute to long-term depression and even social withdrawal (10, 39). This is because communicating with older parents about the death of their child is difficult and can be hurtful and stigmatizing (39). Older adults necessitate continuous care and social support, including old-age security, psychological aid, and grief counselling. Primary care organizations should consistently track, evaluate, and intervene in the activities of older parents with child bereavement, encompassing living needs, mental health, physiological disease, and even spiritual needs. In addition, states should increase benefits (pension and medical insurance) to these older adults and provide social services, especially mental health services.

The highest percentage of older adults with child bereavement was observed in the group with stabilized low depressive symptoms, which is consistent with a previous study (24). This trajectory group had a slow increase in depressive symptoms at first and then a gradual decrease. Depression peaked at one timepoint, and its overall volatility was low. This may be related to individual coping style and resilience. An individual’s adaptability, which is an important coping quality, plays a key role in recovery when they experience adversity. Previous studies (24, 40, 41) have shown that high resilience and positive coping are negative predictors of depressive symptoms and that when individuals face adversity or a major negative life event, it stimulates their protective mechanisms (e.g., resilience) to resist negative emotions, effectively alleviating maladaptation after the loss of a child. Therefore, we explored the characteristics of individuals in the group with low depressive symptoms to provide effective intervention measures to other groups. In addition, we should encourage individuals in this group to continue maintaining a good mental state and ensure that their living needs are met.

### 4.2 Analysis of factors influencing potential categories of depressive symptoms

#### 4.2.1 Demographic factors

The trajectory of bereavement-related depressive symptoms has been found to be associated with residence, with urban older adults with child bereavement being less likely to be in the C3 group than rural older adults. Previous studies (42) have found that place of residence was a predictor of bereavement-related depressive symptoms, with urban older adults having significantly lower depressive symptoms than rural older adults, which is consistent with the results of this study. This observation is primarily attributable to the fact that urban areas are rich in high-quality healthcare resources, which makes it easier for the older adult to access professional psychological interventions (43). In urban areas, a well-developed social security system reduces the financial burden, and a variety of social activities provide adequate emotional support. Rural areas, however, are weaker in terms of medical care, security, and social support, which makes older adults in these areas more prone to high levels of depressive symptoms. The government should pay close attention to the social and psychological needs of the older people with child bereavement in rural areas. On the one hand, it should increase the investment in rural healthcare resources and should actively carry out mental health screening and free medical consultations in these areas; on the other hand, it should improve the social security system in rural areas, increase the standard of old-age pensions, and set up a special relief fund to alleviate their financial burdens. Furthermore, older adults with bereavement in rural regions should be given priority access to psychological services, and volunteers should be organized to care for them, encouraging them to express their life and psychological needs, so as to comprehensively improve the medical, economic, and social conditions of these individuals, to promote their psychological health, and to reduce the risk of depression.

#### 4.2.2 Others factors

In this study, older adults with child bereavement who experienced restless sleep most or all of the time were more likely to be categorized into the C3 group than in the C1 and C4 groups. This indicates a strong association between poor sleep quality and a specific depressive symptom trajectory. Continuous restlessness in sleep may disrupt the normal psychological and physiological rhythms of these older adults, gradually leading to a more severe and complex pattern of depressive symptoms as represented by the C3 group. Sveen et al. (37) also reported that older adults with child bereavement who had severe insomnia showed significant depressive symptoms, which is consistent with our finding and suggests that sleep quality is an important predictor of the trajectory of depressive symptoms. The trajectory of bereavement-related depressive symptoms has also been found to be associated with satisfaction with life, and older adults who are dissatisfied with their life are more likely to be categorized into the C3 group those in the C4 group. Dissatisfaction with life can create a negative psychological environment, which may exacerbate the depressive symptoms of these older adults over time. A study has shown (21) that older parents who have lost their children reported more depressive symptoms and lower life satisfaction than older parents who have not lost their children. The present study further emphasizes that life satisfaction is a significant factor influencing the trajectory of depressive symptoms. A lower level of life satisfaction at the initial stage may set the stage for a more challenging trajectory of depressive symptoms. In addition, the results of this study showed that older adults with child bereavement who self-reported good health were approximately 5.5 times more likely to be categorized into the C4 group than those with poor self-reported health. Self-reported health reflects an individual’s subjective perception of their physical and mental state. A positive self-reported health status may be associated with better coping mechanisms and a more optimistic attitude, which in turn contributes to a more favorable trajectory of depressive symptoms. However, in Ye et al.’s (38) study, self-reported health status was not a factor for depression among older adults with bereavement. This discrepancy might be due to the limited sample source in Ye et al.’s (38) study, which was restricted to three cities in China and may not be well representative of the general population. The co-occurrence of these factors increases the complexity of bereavement-related depression, resulting in higher levels of depression in the C3 group. These findings reveal that the government should prioritize care for older adults with child bereavement who are residing in rural regions and who report poor health. By offering appropriate material needs and personalized health plans that can prevent the development of depression and improve their health conditions and by providing volunteer help to these individuals, the government can better address the situation.

## 5 Limitations

This study has some limitations. First, due to the long-term follow-up of the study cohort, some older adult individuals who had experienced child bereavement were lost to follow-up, resulting in a limited sample size for this study. Although a baseline characteristic comparison analysis was carried out to verify the representativeness of the sample, the validity of the model was, to a certain extent, still restricted. Second, because this study did not incorporate data from every longitudinal data point, the effects of specific alterations in individual health factors on the evolution of bereavement-related depressive symptoms could not be investigated. This limitation restricts our understanding of how these factors interact and contribute to the dynamic nature of bereavement-related depression. Third, key potential factors such as income and time since losing a child were not included. Income can affect access to resources and wellbeing, and the impact of the grieving period varies depending on depressive symptoms. Not including income and time since losing a child may limit our ability to fully capture all relevant influences on depressive symptoms. Finally, due to the long time span, it was difficult to measure the effects of environmental changes and other latent influencing factors. Therefore, in future studies that explore the trajectories of depressive symptoms among older adults who have experienced loss of a child, it is necessary to further reduce the dropout rate and increase the sample size. In addition, efforts should be made to enhance the ability to describe the relationship between depression resulting from the loss of a child and baseline factors and to expand the exploration of other key factors.

## 6 Conclusion

In conclusion, this study identified four different latent categories of depressive symptoms in Chinese older adults who lost a child, revealing the diverse trajectories of depression levels in older adults with child bereavement. The government and medical institutions should develop predictive social security policies and healthcare measures based on the changing characteristics of depression trajectories. In addition, it is recommended that medical institutions prioritize long-term dynamic evaluation for rural older adults with a high level of sleep restlessness, who usually are not satisfied with life and self-report that their health is fragile. Future studies should develop a dynamic evaluation system for different levels of depressive symptoms in older adults with child bereavement in China to better address the depressive symptoms of older adults at different times.

## Data Availability

The raw data supporting the conclusions of this article will be made available by the authors, without undue reservation.
